# The transition of surgical simulation training and its learning curve: a bibliometric analysis from 2000 to 2023

**DOI:** 10.1097/JS9.0000000000001579

**Published:** 2024-05-09

**Authors:** Jun Zhang, Zai Luo, Renchao Zhang, Zehao Ding, Yuan Fang, Chao Han, Weidong Wu, Gang Cen, Zhengjun Qiu, Chen Huang

**Affiliations:** a Department of Gastrointestinal Surgery, Shanghai General Hospital, Shanghai Jiao Tong University School of Medicine, Shanghai, the People’s Republic of China; b The Affiliated Chuzhou Hospital of Anhui Medical University, Anhui, the People's Republic of China

**Keywords:** bibliometric, learning curve, simulation, surgical training

## Abstract

**Background:**

Proficient surgical skills are essential for surgeons, making surgical training an important part of surgical education. The development of technology promotes the diversification of surgical training types. This study analyzes the changes in surgical training patterns from the perspective of bibliometrics, and applies the learning curves as a measure to demonstrate their teaching ability.

**Method:**

Related papers were searched in the Web of Science database using the following formula: TS=[(training OR simulation) AND (learning curve) AND (surgical)]. Two researchers browsed the papers to ensure that the topics of articles were focused on the impact of surgical simulation training on the learning curve. CiteSpace, VOSviewer, and R packages were applied to analyze the publication trends, countries, authors, keywords, and references of selected articles.

**Result:**

Ultimately, 2461 documents were screened and analyzed. The USA is the most productive and influential country in this field. *Surgical endoscopy and other interventional techniques* publish the most articles, while *surgical endoscopy and other interventional techniques* is the most cited journal. Aggarwal Rajesh is the most productive and influential author. Keyword and reference analyses reveal that laparoscopic surgery, robotic surgery, virtue reality, and artificial intelligence were the hotspots in the field.

**Conclusion:**

This study provided a global overview of the current state and future trend in the surgical education field. The study surmised the applicability of different surgical simulation types by comparing and analyzing the learning curves, which is helpful for the development of this field.

## Introduction

Highlights

- Surgical simulation has become popular since 2014, and the annual publications increased dramatically after 2014.
- The United State is the most productive and influential countries, and Aggarwal Rajesh plays a predominant role in promoting collaboration among authors.
- Laparoscopic and robotic have become the mainstream surgical techniques in the field of surgery, while virtue reality and artificial intelligence play an important role in surgical simulation.
- Objective and widely applicable tools, surgical simulation procedures, and high-quality assessment tools are the main directions for future research.

The history of surgical training can be traced back to the 1800s, when surgeons practiced procedures with animals or cadavers^1^. The development of surgical technology has led to continuous changes in surgical training models. With the emergence of laparoscopic technology, apprenticeship-based skill training has become the mainstream form of surgical skill training. Resident physicians participate in surgical procedures as assistants and receive practical training. However, in actual surgery, assistants rarely have the opportunity to perform practical operations, and more often do auxiliary work such as fixing instruments^2^. This training mode has a longer learning curve, requires more time to acquire surgical skills and lacks objective evaluation criteria. Moreover, the emergence of surgical robots leaves us in search of better options. In the last decade, the American College of Surgeons strongly recommends incorporating simulation training into surgical skill training^3^. Simulation training can provide realistic haptic feedback and objective evaluation in nonoperating room environments, reduce training time, and obtain accurate learning curves. Based on the stage of the learning curve, more personalized training can be provided to residents. In addition, acquiring skills from simulation training contribute to improving patient safety. Currently, an increasing number of institutions are developing training programs that include surgical simulation and evaluation tools, which marks a novel revolution in surgical education^4,5^.

Herein, we summarize the changes and developments in surgical simulation training over the past two decades. R package, CiteSpace, and VOSviewer were utilized to analyze the hot topics of surgical training in the past and predicted possible future directions in this field. Moreover, we discussed the learning curve of different simulations and surmised the applicability of simulation to surgical training. We hope to provide valuable insights into the future trends of surgical training through literature, ultimately enabling researchers and surgeons to have a deeper understanding of the current situation in this field and to clarify future development directions. The aim of the bibliometric analyses was to clarify the evolution and importance of surgical training through the analysis of relevant articles published in the field of surgical training, and to demonstrate the superiority of new simulation training techniques and minimally invasive techniques through the learning curves of different surgical training methods.

## Method

### Search strategy

**Figure 1:**
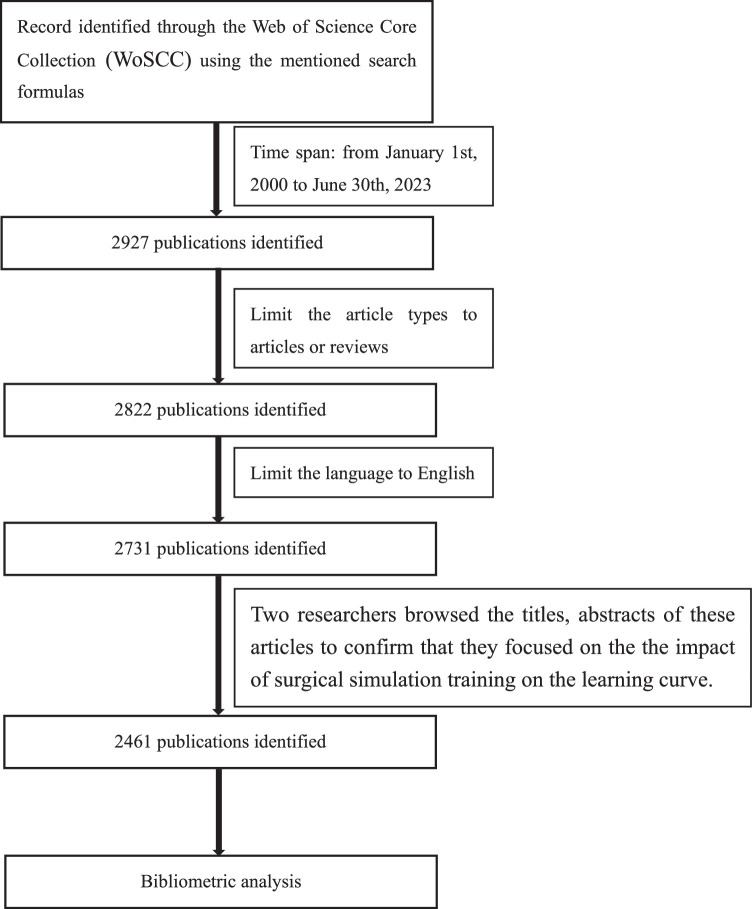
Specific details about the search terms and criteria used for article selection.

The literature retrieval and data were downloaded from the Web of Science Core Collection (WoSCC) database using the following formulas: TS=[(training OR simulation) AND (learning curve) AND (surgical)]. To eliminate the impact of data updates, we limited time from 1 January 2000 to 30 June 2023. The document types were limited to articles or reviews and the document language was limited to English. The search yielded a total of 2927 papers. Two researchers browsed the titles, abstracts of these articles to confirm that they focused on the impact of surgical simulation training on the learning curve. Ultimately, 2461 studies were screened and applied for further analysis in this study (Fig. 1).

### Bibliometric analyzing

The preprocess of data was conducted using Microsoft Excel. All data of the documents were imported into the R4.3.2, CiteSpace (version 6.2.R4) and VOSviewer 1.6.18.

In the study, R packages bibliometrix and ggplot2 were used for data analysis and visualization through R studio. R package is a collection of R codes, data and documentation to provide specific function and solve specific problems through R language. These packages are created by researchers to extend the functionality of R. Users can conduct various statistical analysis, data manipulation, visualization by utilizing these R packages through platform like CRAN (Comprehensive R Archive Network)^6^. Through code reprogramming and collaboration among users, the functionality of R packages is constantly expanding, providing more standardized tools for data analysis and scientific research. R packages bibliometrix and ggplot2 are open-source tools that can be programmed in R studio for conducting bibliometric analysis and presenting comprehensive visualized images. We employed R packages to present publication trend map and keywords heat map based on time series.

CiteSpace is a full-featured visualization software developed by Chen *et al*.^7^, which provides an experimental platform to perform co-occurrence visual network, detect the hotspots and predict the future research direction in a specific field. CiteSpace can analyze the literatures from multiple angles like author, keyword, country, and so on. Briefly, first, we uploaded the files to CiteSpace to eliminate duplicates through duplicates removing functions and save these articles in plain files for further analysis. Second, we selected the node type based on our analysis requirements and visualized the cluster of keywords, co-occurrence map of references and references citation burst map to illustrate the hot directions, observe the research development process and detect topic change trends. The parameters used for analysis were as follows: time span (2000–2023), years per slice (1), selection criteria (g-index: k=25), pruning (Pathfinder), and other settings were set to default value.

VOSviewer is one of the most extensively used tools in bibliometric analysis, which was designed by Nees Jan van Eck and Ludo Waltman in 2009. VOS stands for visualization of similarities, the strong graphic ability makes it suitable for processing large-scale data and presenting it in the form of images^8^. VOSviewer can build a mapping citation data extracted from Web of Science and construct the relationship of networks. As mentioned above, the files were imported into VOSviewer to conduct analysis first. In our study, VOSviewer mainly completed the country and author analysis, keyword co-occurrence and density analysis, the minimum number of occurrences of each country, author and keyword were set to one, two, and five. Then the LinLog and Fractional method of VOSviewer were applied to conduct analysis and modify figures. The size of the nodes and the links between the nodes denote the significance and relationship between nodes, the color of nodes represents its active time, yellow indicates its appearance later, while green indicates earlier.

## Result

### Publication trend analysis

**Figure 2:**
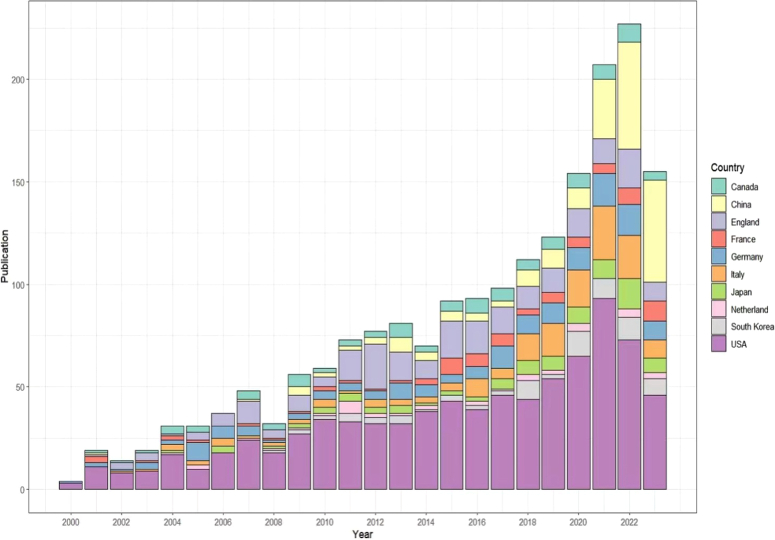
Overview of publication trend of the top 10 countries.

A total of 2461 articles were included in our analysis. The earliest research on surgical simulation training can be traced back to 1991, which is about the changes in the learning curve of laparoscopic cholecystectomy^9^. However, due to the limited number of literatures before 2000, we did not include it in the analysis. Figure 2 shows the publication trend of papers published from 2000 to 2023. The number of publications generally increased with the development of surgery. Before 2008, the number of articles published was relatively small, while the number of articles steadily increased after 2008. Since 2014, the annual publication volume has increased dramatically. The annual publication volume in the past 2 years has exceeded 200, and according to this trend, the number of publications in 2023 will exceed that in 2022, indicating that surgical simulation training has been increasingly valued.

### Country analysis

**Figure 3:**
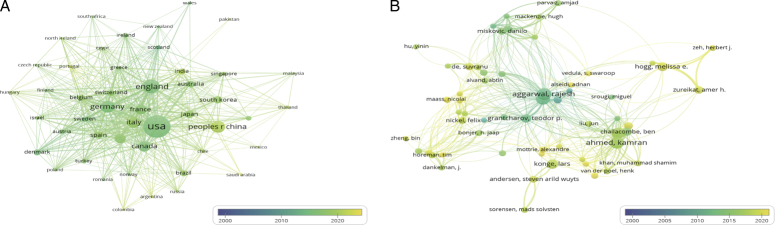
Collaboration map of country(A) and author(B).

To further evaluate the influence of different countries in this field, we analyzed the number of publications and citations. A total of 79 countries have published papers. Table 1 summarizes the top 10 countries with the highest number of articles. The US is the country with the highest number of publications, followed closely by England and China. According to the number of publications, North America, Europe, and East Asia are the most influential regions in the world. The improvement of the learning curve by surgical training was first discovered and published in the US in 1991 and gradually gained attention in the US. As shown in Figure 3A, early articles were mainly published by the United States, and today the US still accounts for a considerable proportion of articles published annually. Moreover, there has been an apparent and qualitative leap in the number of papers published by China in the last five years. After 2020, China and the US accounted for 50% of the total number of annual publications. However, for the average citations, Japan is far ahead of than other countries, while China is the least (Table 1). Figure 3A is a network visualization map of international research collaboration among countries. The size of the node indicates the number of publications, and the number of connections indicates the degree of cooperation with other countries. As the first country in this field, the US has tight collaboration with other countries such as Germany and England.

**Table 1:** List of the top 10 countries.

Ranking	Country	Number of publications	Citation	Average citation
1st	USA	818	21 567	26.36
2nd	England	329	10 094	30.68
3rd	China	274	2717	9.92
4th	Germany	235	5084	21.63
5th	Italy	190	3840	20.21
6th	Canada	166	3923	23.63
7th	France	154	4282	27.81
8th	Japan	93	4419	47.51
9th	South Korea	91	1568	17.23
10th	Spain	81	1959	24.19

### Journal and co-cited journal analysis

A total of 615 journals contributed 2461 papers. Table 2 presents the top 10 journals that have published the most papers. *Surgical endoscopy and other interventional techniques* is the leading journal with 198 publications, accounting for 8.05% of all publications, which is followed by *Journal of Surgical Education*, *Journal of Endourology*, and *Annals of Surgery*. Among the 10 journals with the highest number of related articles published, 7 are all from the US, which further demonstrates the influence of the US in this field. The citations of a journal are the number of citations for papers published in the journal. As depicted in Table 2, *Surgical Endoscopy and other Interventional Techniques* is the most cited journal and is also the most published journal in the field, demonstrating that this is an influential journal in the field of surgical simulation. Next is *Annals of Surgery* and *Journal of Urology*, which is the journals with the highest average citations, suggesting its authority in this field.

**Table 2:** List of top 10 journals and co-cited journals.

Ranking	Journal	Count	IF (2022)	Country	JCR (2022)	Co-cited journal	Citation	IF (2022)	Country	JCR (2022)
1st	Surgical endoscopy and other interventional techniques	198	3.1	Switzerland	Q2	Surgical endoscopy and other interventional techniques	5580	3.1	Switzerland	Q2
2nd	Journal of Surgical education	75	2.9	USA	Q1	Annals of Surgery	2909	9	USA	Q1
3rd	Journal of Endourology	42	2.7	USA	Q3	Journal of Urology	2444	6.6	Netherlands	Q1
4th	Annals of Surgery	35	9	USA	Q1	American Journal of Surgery	1784	3	USA	Q2
5th	American Journal of Surgery	32	3	USA	Q2	Journal of Surgery Education	1430	2.9	USA	Q1
6th	Journal of Robotic surgery	28	2.3	USA	Q2	Bju International	1092	4.5	England	Q1
7th	International Journal of Medical Robotics and Computer Assisted Surgery	28	2.5	England	Q3	Hernia	1018	2.44	France	Q2
8th	Journal of Laparoendscopic & Advanced Surgical Techniques	27	1.3	USA	Q3	Journal of Endourology	785	2.7	USA	Q3
9th	World Neurosurgery	27	2	USA	Q3	European urology	777	23.4	Netherlands	Q1
10th	Bju International	26	4.5	England	Q1	British Journal of Surgery	704	9.6	USA	Q1

### Author and co-cited author analysis

These articles were jointly contributed by 13 225 authors, among which, Ahmed Kamran and Aggarwal Rajesh are the most productive authors, followed by Darzi Ara, Dasgupta Prokar, and Konge Lars. Figure 3B shows the collaboration map, which reflects the number of publications, publication time, and collaboration between authors. The average publication time of Aggarwal Rajesh’s article is earlier than that of Ahmed Kamran. Meanwhile, the map illustrates that Aggarwal Rajesh plays a predominant role in promoting collaboration among authors.

Co-cited author is that two authors are both cited by one paper simultaneously. An author is cited more frequently, indicating that the content and results of the articles are more convincing. As illustrated in Table 3, the most frequently co-cited author is Aggarwal Rajesh, who is also one of the most productive authors. The analysis of authors and co-cited authors provides the information on the most influential author in this field.

**Table 3:** List of top 10 productive and co-cited authors.

Ranking	Author	Count	Co-cited author	Count
1st	Ahmed, Kamran	25	Aggarwal, Rajesh	1713
2nd	Aggarwal, Rajesh	25	Darzi, Ara	1343
3rd	Darzi, Ara	23	Grantcharov, Teodor P	1211
4th	Dasgupta, Prokar	21	Ahmed, Kamran	883
5th	Konge, Lars	15	Dasgupta, Prokar	695
6th	Hogg, Melissa E	14	Aggarwal, R	560
7th	Grantcharov, Teodor P	12	Miskovic, Danilo	541
8th	Challacombe, Ben	11	Hanna, George B	539
9th	Zureikat, Amer H	10	Besselink, Marc g	518
10th	Miskovic, Sanilo	9	Challacombe, Ben	514

### Keyword analysis

**Figure 4:**
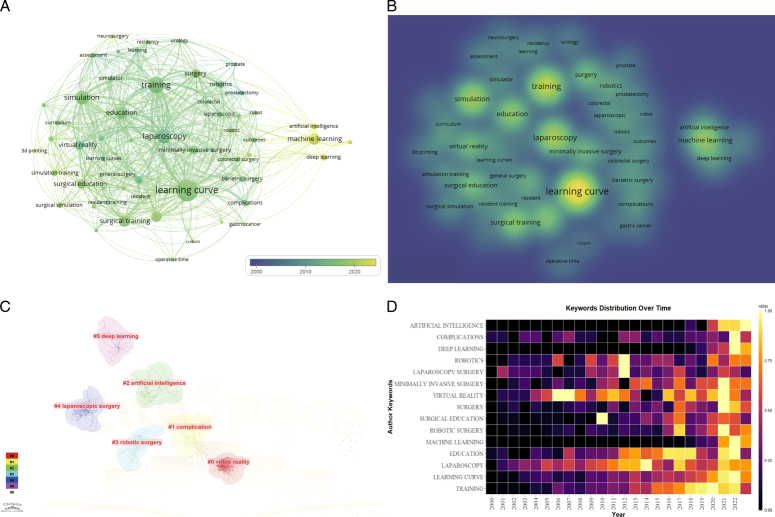
(A) Visualization map of the keyword analysis. (B) A density map of keywords. (C) Network map of keyword clusters analysis. (D) Timeline view of keyword.

Keyword analysis can well reflect the research hotspots in the field of changes in learning curves caused by surgical training. Before analysis, we merged keywords with similar meanings and generated a list of the top 20 most frequent keywords and a keyword occurrence map to analyze the directions and frontiers of surgical simulation. Table 4 shows the frequency of the top 20 keywords. Figure 4A is the occurrence map generated by VOSviewer. The colors of the nodes represent the average year in which the keyword appears, and the color from purple to yellow indicates time from far to near. In early years, the keywords ‘laparoscopy, minimally invasive surgery’ appear more in early time, keyword ‘robotics’ appears slightly later. ‘AI, deep learning, and machine learning’ have appeared in recent years. The above changes indicate the revolution in research hotspots over the past 20 years. Figure 4B is a density map, which can also intuitively reflect the frequency of keywords appearing.

**Table 4:** List of the top 20 keywords.

Ranking	Keyword	Count	Ranking	Keyword	Count
1st	Learning curve	1100	11th	Complication	226
2nd	Surgery	570	12th	Validation	180
3rd	Training	437	13th	Impact	157
4th	Laparoscopy	362	14th	Machine learning	151
5th	Education	351	15th	Robotic surgery	137
6th	Performance	331	16th	Minimally invasive surgery	122
7th	Simulation	321	17th	Model	111
8th	Outcome	308	18th	Operating room	109
9th	Experience	288	19th	Cancer	109
10th	Skills	238	20th	Acquisition	107

To further reflect the research direction with the development of technology and the evolution process of surgical simulation, cluster analysis was performed using CiteSpace. Six clusters were obtained by the keyword clustering function of CiteSpace, each represented a research direction (Fig. 4C). The label of each cluster is the most representative word in each cluster: cluster 0 (represented by simulation), cluster 1 (represented by complication), cluster 2 (represented by machine learning), cluster 3 (represented by robotic surgery), cluster 4 (represented by laparoscopic surgery), and cluster 5 (represented by learning curve).

‘Burst’ means that this word appears frequently and lasts for a period of time. Keyword burst analysis could reflect the transition of popular directions of the field during certain periods and predict the future direction. Figure 4D presents the top 15 keywords with the strongest burst. In Figure 4D, darker colors indicate a lower frequency of keyword occurrences, while brighter colors indicate a higher frequency of keyword occurrences. ‘VR’ began to arise in 2005 and reached its peak in 2006 and 2007, attracting much attention from people. Laparoscopy began to be popular in 2005 and has gradually become widely known and was the most popular research direction. Currently, virtual reality is still a research hotspot. In terms of burst time, ‘AI’, ‘deep learning’, and ‘robotics surgery’ began to explode in recent years, implying that the research hotspots have gradually shifted to the field of artificial intelligence (AI).

### Reference analysis

The frequency of co-citation article is often associated with high academic value. Highly cited article usually possesses important reference value and guides significance for subsequent research. The ten most cited references are listed in Table 5, all of which were published before 2010, indicating that papers published in recent years still need more time to be verified and cited. The most cited paper is published in *Annal of Surgery* ^10^. The second most cited paper was contributed by Grantcharov in 2004^11^. The centrality of the above two articles is relatively high, indicating that they have a significant impact on subsequent articles. The third most cited article is ‘Evaluation of the learning curve in laparoscopic colorectal surgery: comparison of right-sided and left-sided resections^12^’.

**Table 5:** List of top 10 cited references related to surgical simulation.

Ranking	Year	Author	centrality	Title	Citation
1st	2002	Seymour Ne	0.10	Virtual reality training improves operating room performance: results of a randomized, double-blinded study	173
2nd	2004	Grantcharov tp	0.08	Randomized clinical trial of virtual reality simulation for laparoscopic skills training	113
3rd	2005	Tekkis pp	0.01	Evaluation of the learning curve in laparoscopic colorectal surgery: comparison of right-sided and left-sided resections	106
4th	2004	Dindo D	0.01	Classification of surgical complications: a new proposal with evaluation in a cohort of 6336 patients and results of a survey	94
5th	2006	Reznick Rk	0.01	Teaching surgical skills–changes in the wind	80
6th	2002	Gallagher Ag	0.01	Virtual reality as a metric for the assessment of laparoscopic psychomotor skills. Learning curves and reliability measures	73
7th	2003	Grantcharov tp	0.12	Learning curves and impact of previous operative experience on performance on a virtual reality simulator to test laparoscopic surgical skills	64
8th	2001	Schlachta Cm	0.01	Defining a learning curve for laparoscopic colorectal resections	61
9th	1999	Bridges M	0.05	The financial impact of teaching surgical residents in the operating room	59
10th	2004	Fried Gm	0.00	Proving the value of simulation in laparoscopic surgery	58

**Figure 5:**
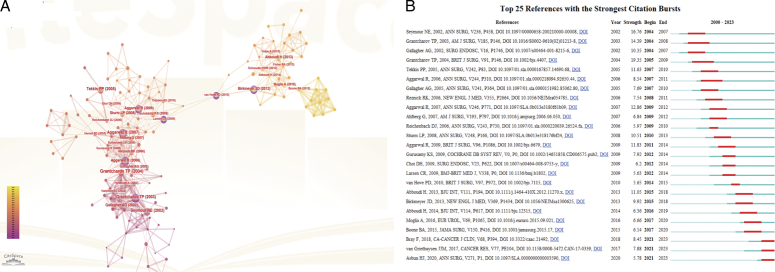
(A) Visualization map of the keyword analysis. (B) Citation bursts of references related to ferroptosis in cancer.

Figure 5A presents the occurrence map of references. The node surrounded by a purple ring is the reference that has high centrality. A review published by van Hove PD in 2010 presented the highest centrality (0.31), which summarized several current surgical skill assessment methods^13^. This article objectively analyzed the validity and reliability of existing methods such as VR simulator and global rating scales. It concluded that most assessments could well reflect the training results. Appropriate and standardized evaluation methods should be selected for different application scenarios. Figure 5A also shows the bridging role of this article in different research directions of the surgical simulation field. Article ‘Effect of virtual reality training on laparoscopic surgery: randomized controlled trial’ exhibits a centrality of 0.3, which focused on the laparoscopic salpingectomy^14^. VR training can significantly improve operative skills and shorten the learning curve.

The top 25 references with citation bursts are shown in Figure 5B. The blue line represents the time span, while the red line represents the time period of the reference burst. The top ranked article based on citation burst ranks second according to the citations. Moreover, this article also has the longest citation time. The other two articles with a citation burst time of 4 years are both about the learning curve in surgical training of laparoscopic surgery, suggesting the popularity of laparoscopy^12,15^.

## Discussion

In recent decades, the field of surgery has undergone rapid development. Surgical simulation training has demonstrated the potential to enhance surgical skills, mitigate risks, and has become a focal point in recent years. A prerequisite for residents embarking on real patient procedures is a comprehensive grasp of surgical training^16^. In 1991, Peters conducted prospective research on surgical simulation and found that preoperative biliary training can significantly improve operative efficiency and exhibit a significant learning curve^9^. In the same year, the USA emphasized for the first time the importance of preoperative simulation training. This marked the inception of surgical training, a continuum that persists to the present day^17^. Figure 2 presents the growth trend of publication related to surgical simulation. The annual growth trend can be divided into two discernible periods, including steady stage and rapid development stage. In the steady development phase (2000–2014), annual publication exhibited consistent growth, notwithstanding minor fluctuations. After 2014, the field of surgical training experienced a rapid development stage, marked by a dramatic increase in annual publication volume. This surge signified an escalating interest and emphasis on research in surgical training. There may be attributed to multiple reasons. First, the rising popularity of laparoscopy, attributed to its minimally invasive nature and reduced postoperative pain^18^, is can be reflected in Figure 4D. Second, compared to open surgery, laparoscopic surgery is more difficult. The inherent complexity of laparoscopic surgery necessitates extensive training, particularly for young doctors lacking experience, accentuating the growing awareness of the pivotal role of surgical training in ensuring procedural safety.

In terms of national distribution, the landscape of research on the surgical simulation training exhibits an evident imbalance. The top 10 countries account for a substantial majority, with 2431 articles, representing 98.78% of the total publications. Notably, the majority of research in this field is conducted by developed countries, resulting in a huge gap between developing and developed countries. This disparity is underscored in Tables 1 and 6, where China stands as the sole developing country, while nine out of the top 10 countries with the highest publications are developed. The US, contributing almost one-third of related articles, holds a dominant position among the top countries. The prominence of US in publication output may be attributed to their advanced hardware and software infrastructure, coupled with substantial financial support^19^. Additionally, the well-established and standardized system in the field of surgical training in the United States likely contribute to its leading position. However, recent trends indicate a declining proportion of annual publications from the United States, contrasting with the upward trajectory observed in China (Fig. 2). This shift may be associated with the expanding demand for surgical expertise in China, capturing increased attention from doctors and researchers^20^. Furthermore, there is a continuous elevation in the international recognition of surgical simulation training. The Chinese researchers is inclining towards integrating international practices, thereby fostering the growth of pertinent research. From a citation perspective, the proficiency of USA, England, and Japan in producing high-quality articles can be ascribed in part to their enduring collaborations and exchanges with other nations. The high average citations of these countries are at the forefront, denoting their substantial influence and authoritative standing in research. These observations underscore the pivotal role of international cooperation in fostering research competitiveness. Lack of international cooperation will make research results less convincing, as prospective studies from multiple centers and regions can better demonstrate the effectiveness of surgical training and obtain higher-level clinical evidence to guide clinical practice. International collaboration not only provides a broader platform for the exchange and sharing of ideas but also fosters mutual learning, allowing for the collision and emergence of novel concepts. Notably, China exhibits the lowest average citation among the top countries. This may be caused by various reasons. First, China’s research in this field started relatively late, and it takes longer for many studies to obtain more authoritative results. Second, most papers in China were published in recent years, and the results need more time to be further verified and cited. Third, it may be due to limited exchanges between China and other countries, as reflected in Figure 3A. Consequently, it is imperative for Chinese researchers to strengthen international collaborations and establish a standardized research system to generate higher-quality contributions. International cooperation can provide a larger platform for the exchange of ideas and learning, promoting the development of the field. Thus, international cooperation is encouraging. Encouraging international cooperation emerges as a crucial strategy for fostering innovation and advancing the field.

**Table 6:** List of top 10 productive institutions.

Ranking	Institution	Count	Citations
1st	Univ Toronto	64	1816
2nd	Univ London Imperial Coll Sci Technol and Med	40	2126
3rd	Johns Hopkins Univ	33	786
4th	Mayo Clin	32	545
5th	Univ Pittsburgh	31	1131
6th	Kings Coll London	28	852
7th	Harvard Med Univ	26	434
8th	Heidelberg Univ	25	541
9th	Mcgill Univ	25	612
10th	Stanford Univ	25	438

As for journal, the Impact Factor (IF) and Journal Citation Reports (JCR) are potent indicators to judge the impact and authority of a journal in particular field^21^. Higher IF signifies publications in the journal garner more citation, underscoring its prominence within the respective field. JCR divides journals into four levels based on their impact factors, namely Q1, Q2, Q3, and Q4^19^. By assessing the volume of publications and citations in the journal, we can identify which journals are the core journals in the field. As illustrated in Table 2, *Surgical endoscopy and other interventional techniques*, *Journal of Surgical Education*, and *Journal of Endourology* hold the top three positions, focusing on the forefront of surgical advancements and surgical education. Notably, *Surgical endoscopy and other interventional techniques* has published most papers including systematic reviews and retrospective studies on surgical training, which also received the most citations, indicating that this journal has a significant influence in this field. Among the top 10 journals, *Annal of Surgery* boasts the highest impact factor in the top and have considerable citations. The journal features articles encompassing systematic reviews and prospective analyses, indicative of a high level of evidence. In the table of top journals and co-cited journals, most journals are from USA, further illustrates the dominance of USA. Despite the recent surge in research output from China, its international influence remains comparatively modest. Hence, it is imperative for China to increase the publication of high-quality articles and establish journals that wield international influence.

Rajesh Aggarwal form Imperial College London emerges as the most prolific author, securing the top position in co-citation rankings owing to his significant contributions to the advancement of surgical simulation. In 2006, Rajesh Aggarwal published an article titled ‘Technical-Skills T raining in the 21th Century’ in *The New England Journal of Medicine* ^22^. Within this article, Aggarwal advocated for the integration of virtue reality (VR) into surgical education, positing that this innovative approach could enhance clinical performance and elevate professionalism. Expanding on this theme in 2009, Aggarwal underscored the potential application of surgical simulation in *the BMJ* ^23^, emphasizing the necessity for future research to delineate the intricate connections between virtual reality, simulated operating rooms, and real-world environments—a perspective that profoundly propels the evolution of surgical simulation. In the same year, a comparative study conducted by Aggarwal, published in *European Urology*, demonstrated that simulator training significantly enhanced the performance of primary urology residents during their initial laparoscopic procedures^24^. Ahmed Kamran from King’s College London is another scientist who published the most articles. Several high impact factor articles published by Ahmed are all about the beneficial role of surgical simulation training in urology^25–27^. His contributions encompass multiple evaluations and systematic reviews, consistently affirming the effectiveness of simulation training in abbreviating the learning curve for urologists. Kamran advocates for the integration of simulation into surgical curricula. Notably, British researchers, exemplified by Aggarwal and Kamran, have conducted compelling studies, significantly advancing the field of simulation training.

Influential literature can reflect the hot research directions in this field to some extent. The most cited article first validated the role of VR in training surgical residents and reducing the incidents. In this study, Seymour *et al*. found that virtual reality training could improve the operating performance of surgical residents in laparoscopic cholecystectomy by comparing the operative error of the VR-training group and non-VR-training residents. The result showed that the speed of surgery increased by 29% and fewer errors were made during surgery. This landmark study first introduced VR technology into surgical education and achieved satisfactory results. Moreover, the second most cited literature is also about the role of VR in laparoscopic surgery training, whose results are similar to the those of the above article. The third most cited article analyzed the learning curve of laparoscopic left and right sides colonic resections. The results of the learning curves showed that the laparoscopic-open surgery conversion rate and median operating time both improved with operative experience, while there was no difference in the postoperative complications and readmission rates. However, different results have been obtained in other studies^28,29^, which may be due to the multiple influencing factors of laparoscopic surgery. Based on the result of keywords analysis and reference analysis, discernible trends in the realm of surgical simulation research emerge. Predominant research directions include ‘Laparoscopic surgery’, ‘Robotic surgery’, ‘VR’, and ‘AI’. Laparoscopic and robotic surgeries stand out as the prevailing methods in surgical procedures, encompassing the majority of interventions. VR holds a pivotal role in the domain of surgical simulation training, a fact substantiated by a multitude of studies affirming its efficacy in enhancing surgical training. Furthermore, AI emerges as a crucial component, offering objective evaluation criteria for appraising the efficacy of surgical training protocols.

Compared to open surgery, laparoscopic surgery presents increased complexity and heightened surgical risks. Experienced surgeons can master laparoscopic surgery well, while less-experienced practitioners, such as young doctors may lack the requisite skills to ensure surgical safety. Thus, the USA also emphasized for the first time the importance of preoperative simulation training in 1991^17,30^, which opened the prelude to surgical simulation training. At present, laparoscopic surgery has emerged as the gold standard for treating various diseases such as gastric cancer and liver cancer, owing to its minimally invasive and causes less postoperative pain^18^. Currently, many studies have been conducted to elucidate the learning curve and safety of laparoscopic surgery training. A study conducted by der Pole *et al*.^31^ evaluated the learning curve of laparoscopic hemihepatectomy, retrospectively analyzing 159 hemihepatectomy cases from 2003 to 2015. The results demonstrated the learning curve of 55 cases for conversions, affirming the feasibility of laparoscopic hemihepatectomy but emphasizing the need for substantial training prior to implementation. Another study in 2020 scrutinized the learning curve of laparoscopic pancreatoduodenectomy, a challenging surgical procedure^32^. Researchers identified distinct learning curve based on different endpoints, with surgical time and severe postoperative complications stabilizing after 25 cases. When concerning conversion rating, 40 cases are needed required to achieve a stable state. These findings provide valuable insights for designing innovative laparoscopic surgical training.

Robotic surgery, particularly with the da Vinci robot, has emerged as a hotspot in the field of surgery over the past 5 years, representing a milestone in surgical simulation training. In the past decade, the number of robotic-assisted laparoscopies has increased exponentially, and they have been widely used in the departments of urology and general surgery^33,34^. Compared to laparoscopy, the 3D imaging system and ‘wrist-like’ robotic arm improve the precision and stability of robotic surgery^35^. Despite its stability, robotic surgery is more complex and demands heightened proficiency for surgeons. Currently, numerous studies from multiple centers have attested to the safety and feasibility of robot surgery training^36^. In a recent study, 15 surgeons across seven centers underwent training programs, incorporating online video banks and robot simulations, and performed 275 robotic pancreatoduodenectomies in total. The learning curve exhibited an inflection point at 22 cases concerning operating time, delineating the initial and subsequent phases. The median operative time in the second stage was 52 min shorter than that of the first stage, reflecting a condensed learning curve with positive outcomes. The results demonstrated the effectiveness of the robotic surgery simulation in enhancing surgeon proficiency. Reports by Park *et al*.^37^ on 89 laparoscopic and robotic low anterior resections, and studies by Melich *et al*.^38^ covering 106 laparoscopic and 92 robotic resection surgeries, suggesting similar learning curves of laparoscopic and robotic surgery. The inaugural Consensus Meeting on European Robotic Training in 2020 established a basis for a validated and standardized training program, emphasizing the necessity for rigorous and reliable simulation training prior to real-world surgical interventions^39^.

Based on the outcomes derived from our keyword burst and reference analysis, ‘VR’ emerges as a recurrent and pivotal direction, representing a crucial technique in surgical simulation. In 1993, VR was reported for the first time to benefit surgeons in learning anatomy, and realism and immersion of VR are beneficial for skill acquisition^40^. VR facilitates the accurate replication of laparoscopic surgery scenarios, enabling practice beyond the confines of the operating room^41^. Many systematic reviews and meta-analyses have already underscored the efficacy of VR in steepening the learning curve, surpassing traditional training^42–44^. However, disparities emerge when comparing the learning curves of surgeons with different levels of experience. Studies conducted by Aggarwal and Grantcharov found that more experienced surgeons exhibit a steeper learning curve^15,45^. These investigations evaluated the effectiveness of VR simulation by comparing the number of repetitions required for the learning curve to reach a stable state between different groups. The median number of repetitions for untrained surgeons was seven, while for experts, it was two. Conversely, studies by Moore and Hassan yielded disparate findings^44,46^. They illustrated that novices improved faster in contrast to the more experienced surgeons, which indicated that early incorporation of VR simulation proves advantageous for surgeons to enhance their surgical skills. However, it is noteworthy that VR simulation in laparoscopic training remains in the experimental stage. Despite the potential benefits, the predominant focus of surgical training continues to be on apprenticeship, necessitating prolonged learning periods. Further research is imperative to ascertain whether skills gained from VR training can ensure patient safety in surgical interventions and to determine the optimal integration of this technology into the training continuum.

AI presents the capability to furnish personalized feedback for surgical trainees^47^. Employing machine learning, AI can learn from databases and render informed judgments, while deep learning uses multilayer artificial neural networks for fully automated analysis of data, with the ability to learn autonomously^48^. By integrating different AI methods, the results of surgical training can be objectively reflected^49^. Moreover, AI offers distinct advantages over subjective assessment by delivering a more standardized and reliable assessment. Through machine learning algorithms, AI scrutinizes various data such as force measurements in simulated training, to provide more objective evaluations of learning curves^50,51^. Bisssonnette *et al*.^52^ introduced support vector machines to evaluate the proficiency level of surgical training in VR. And AI-defined novel metric established an evaluation system that capable of discerning different levels of simulated training, achieving an impressive accuracy rate of 97.6. This underscores the potential of AI in evaluating surgical simulation. Similarly, Hung *et al*.^53^ applied machine learning to process the collected data from robotic surgery, facilitating an assessment the effectiveness of simulation training. In addition, many other AI strategies have been proposed, but most of them have not yet been applied in clinical practice. Once validated, this method holds the promise of delivering a standardized and objective assessment framework for surgical simulation.

Amidst the COVID-19 pandemic, the closure of conventional simulated modalities has promoted the expansion of simulation resources for surgical training^54^. The increase in demand has catalyzed advancements of the field, leading to the development of various VR simulators and assessment tools. As an educational tool, simulators provide learners an opportunity to acquire technical skills in the early stages, thereby mitigating the learning curve. This study briefly examines the educational efficacy of prevalent simulation training methods, specifically focusing on learning curves. Future research endeavors will consider to establish a competency benchmark for these training and evaluation tools, exploring ways to seamlessly translate the skills acquired through simulation training into real-world applications within the operating room. This pursuit is crucial for refining the assessment methodologies and enhancing the overall effectiveness of simulation resources in surgical training.

There are some limitations in this study. First, only publications in Web of Science were included in this study exclusively, many articles in other database may be missed due to the single source. Besides, only English articles were selected to analyze, which may cause some deviation in the results. Second, articles published in later time may receive a lower number of citations and cannot reflect the impact of an article. This study mainly focuses on highly cited articles, which may have overlooked some potentially influential articles in the future. Third, the selection of the article is further edited by researchers to exclude literature unrelated to the research purpose, which may lead to some manual errors. Fourth, it is essential to note that this analysis does not encompass a retrospective comparative examination of the included studies. This limitation primarily stems from the current absence of standardized, objective, and universally applicable tools for measuring the progress of trainees’ surgical competence across various surgical procedures. Additionally, the absence of high-quality tools to assess the effectiveness and reliability of existing simulators further complicates comprehensive retrospective analyses.

## Conclusion

In this study, CiteSpace and other software were used to visualize the developmental trends and hotspots in this field. The results demonstrated that VR and AI will continue to be the focus in the foreseeable future and lead to a climax. Robot surgery and laparoscopic surgery will become the mainstream surgical methods in the future. The learning curve suggested that novel simulation programs were suitable for current surgery training. In general, this study provides a clear present situation of surgical training and can serve as a foundation for future research.

## Ethical approval

None.

## Consent

None.

## Sources of funding

This article was fundamentally supported by the National Natural Science Foundation of China (No.82072662), Shanghai Municipal Education Commission—Gaofeng Clinical Medicine Grant Support (2019142), Shanghai three-year action plan to promote clinical skills and clinical innovation in municipal hospitals (SHDC2020CR4022), and the 2021 Shanghai ‘Rising Stars of Medical Talent’ Youth Development Program: Outstanding Youth Medical Talents.

## Author contribution

J.Z., Z.L.: conceptualization, data curation, validation, and writing original draft; R.Z. and Z.D.: investigation, methodology, validation, and writing original draft; Y.F. and C.H.: formal analysis, software, and validation; W.W.: validation, visualization, and writing review and editing; G.C.: validation and writing review and editing; Z.Q.: validation and writing review and editing; C.H.: conceptualization, data curation, funding acquisition, project administration, resources, supervision, validation, and writing review and editing.

## Conflicts of interest disclosure

The authors declare that there is no conflicts of interests regarding the publication of this paper.

## Research registration unique identifying number (UIN)

1. Name of the registry: not applicable.
2. Unique identifying number or registration ID: not applicable.
3. Hyperlink to your specific registration (must be publicly accessible and will be checked): not applicable.

## Guarantor

Chen Huang. E-mail: huangchen0204@sjtu.edu.cn

## Data availability statement

The data in this study are available in the public domain and are not confidential. All data can be obtained by contacting the corresponding author: huangchen0204@sjtu.edu.cn with the scientific purpose.

## Provenance and peer review

None.
